# *Gabrb2*-knockout mice displayed schizophrenia-like and comorbid phenotypes with interneuron–astrocyte–microglia dysregulation

**DOI:** 10.1038/s41398-018-0176-9

**Published:** 2018-07-17

**Authors:** Rigil K. Yeung, Zheng-Hua Xiang, Shui-Ying Tsang, Rui Li, Timothy Y. C. Ho, Qi Li, Chok-King Hui, Pak-Chung Sham, Ming-Qi Qiao, Hong Xue

**Affiliations:** 1 Division of Life Science, Applied Genomics Center and State Key Laboratory of Molecular Neuroscience, Hong Kong University of Science and Technology, Clear Water Bay, Hong Kong, People’s Republic of China; 2 Department of Neurobiology, Second Military Medical University, Shanghai, People’s Republic of China; 3 Department of Psychiatry, Li Ka Shing Faculty of Medicine, University of Hong Kong, Hong Kong, People’s Republic of China; 4 Shandong University of Traditional Chinese Medicine, Shandong, People’s Republic of China

## Abstract

Intronic polymorphisms of the GABA_A_ receptor β_2_ subunit gene (*GABRB2*) under adaptive evolution were associated with schizophrenia and reduced expression, especially of the long isoform which differs in electrophysiological properties from the short isoform. The present study was directed to examining the gene dosage effects of *Gabrb2* in knockout mice of both heterozygous (HT) and homozygous (KO) genotypes with respect to possible schizophrenia-like and comorbid phenotypes. The KO mice, and HT mice to a lesser extent, were found to display prepulse inhibition (PPI) deficit, locomotor hyperactivity, stereotypy, sociability impairments, spatial-working and spatial-reference memory deficits, reduced depression and anxiety, and accelerated pentylenetetrazol (PTZ)-induced seizure. In addition, the KO mice were highly susceptible to audiogenic epilepsy. Some of the behavioral phenotypes showed evidence of imprinting, gender effect and amelioration by the antipsychotic risperidone, and the audiogenic epilepsy was inhibited by the antiepileptic diazepam. GABAergic parvalbumin (PV)-positive interneuron dystrophy, astrocyte dystrophy, and extensive microglia activation were observed in the frontotemporal corticolimbic regions, and reduction of newborn neurons was observed in the hippocampus by immunohistochemical staining. The neuroinflammation indicated by microglial activation was accompanied by elevated brain levels of oxidative stress marker malondialdehyde (MDA) and the pro-inflammatory cytokines tumor necrosis factor-alpha (TNF-α) and interleukin-6 (IL-6). These extensive schizophrenia-like and comorbid phenotypes brought about by *Gabrb2* knockout, in conjunction with our previous findings on *GABRB2* association with schizophrenia, support a pivotal role of *GABRB2* in schizophrenia etiology.

## Introduction

Schizophrenia is a multifactorial disease that results from interactions between genetic and environmental factors. The strength of genetic factors is underlined by the rise of lifetime risk of the disease from just below 1% in the general population to over 40% in monozygotic twin studies^1^, leading to extensive searches for the genetic basis of the disease. It was first proposed in the early 1970s that inadequacy of tonically active inhibitory GABAergic neuronal activity relative to excitatory neuronal activity initiates the disease^2^, and evidence supports the etiological participation of GABAergic neurons including parvalbumin-containing ones in the hippocampus^3–5^. At present, neural elements that are recognized to play important roles in schizophrenia include contribution of dopaminergic neurotransmission dysfunction to the genesis of psychotic symptoms and abnormalities of neuronal connectivity possibly involving interneurons^6^.

The first identification of a GABAergic pathway gene as a robust schizophrenia candidate gene was obtained by us based on the association of intronic single nucleotide polymorphisms (SNPs) in a 3551-bp segment in the vicinity of the AluYi6AH-151 insertion in the GABA_A_ receptor β_2_ subunit gene (*GABRB2*) with schizophrenia in Chinese^7^, and later confirmed in other populations^8,9^. Genotype-dependent expression and alternative splicing of the *GABRB2* transcript gave rise to reduced gene expression as well as decreased long-to-short isoform ratio of the β_2_ subunit in postmortem schizophrenic brains, and GABA_A_ receptors carrying the long isoform were positively selected, and more quickly fatigued electrophysiologically than those carrying the short isoform upon repeated stimulation^10–13^. Gene expression was subjected to epigenetic regulations, including partial maternal imprinting, which were perturbed in schizophrenia^14,15^. These findings established not only *GABRB2* as a robust candidate gene, but also a chain of correlations leading from genotypes to alternative splicing and electrophysiological alteration. Moreover, the schizophrenia-associated genotypes of *GABRB2* in the AluYi6AH-151 region were found to be associated with bipolar disorder^16^, heroin addiction^17^, and both positive symptoms in schizophrenia patients and altruism in normal subjects^18^.

More recently, genome-wide association studies (GWAS) identified 108 genetic loci related to schizophrenia that did not include *GABRB2*^19^. On the other hand, when 40 polymorphisms in 12 ‘top’ candidate genes for schizophrenia including *AKT1*, *COMT*, *DAO*, *DRD2*, *DRD4*, *DTNBP1*, *GABRB2*, *IL1B*, *MTHFR*, *PPP3CC*, *SLC6A4*, and *TP53* with familial association data were subjected to meta-analysis, only the disease association of rs1816072 upstream of AluYi6AH-151 in *GABRB2* remained significant after correction for multiple testing^20^. In view of this, the objective of the present study was to examine the relationship between the GABA_A_ β_2_ subunit gene dosage and possible schizophrenia-like symptoms in an animal model. Previously, homozygous *Gabrb2*-knockout mice were found to display normal body weight, elevated level of locomotor activity and loss of more than 50% of total GABA_A_ receptors^21^. In addition, they exhibited reduced duration of loss of the righting reflex due to alcohol and sleep-time induced by GABA_A_ receptor ligands^22^, outer hair cell dysfunction in the cochlea^23^, as well as a novel form of inhibitory synaptic plasticity in the cerebellum^24^. In the present study, *Gabrb2*-knockout mice of both homozygous (KO or *Gabrb2*^−/−^) and heterozygous (HT or *Gabrb2*^+/^^−^) genotypes were compared to wild-type (WT or *Gabrb2*^+/+^) mice regarding the possible presence of schizophrenia-like phenotypes. The results obtained provided evidence for a *GABRB2*-origin of schizophrenia.

## Materials and methods

### Animals

*Gabrb2* HT transgenic mice (C57BL/6-129/SvEv hybrid)^21^ supplied by Taconic Farms, Inc. (New York) were bred to yield WT, HT, and KO mice that were weaned at week-3, genotyped using primers specific for the *Gabrb2* and *Neo* genes (Supplementary Methods) and housed in groups of four to six with water and food ad lib, with a 12-h light cycle from 08:00 to 20:00. All animal experiments were pre-approved by the Animal Ethics Committee of HKUST and conducted in accordance with The Code of Practice for Care and Use of Animals for Experimental Purposes. The Code follows international guidelines of animal welfare and is approved by the Department of Health and the Fisheries and Conservation Department of HKSAR.

### Antibodies and immunofluorescent reagents

ELISA kits for TNF-α and IL-6 were obtained from Invitrogen, USA. Reagents for immunohistochemical analysis included primary antibodies against NeuN (1:500, monoclonal, clone A60; EMD Millipore, MA), GFAP (1:500, rabbit polyclonal; Boster Biological Technology, CA), DCX (1:200, goat polyclonal; Santa Cruz Biotechnology, TX), Iba1 (1:500, rabbit polyclonal; Wako, Japan), and parvalbumin (PV, 1:3000, rabbit polycolonal; Proteintech, IL). The fluorescence-labeled secondary antibodies FITC-conjugated donkey anti-mouse IgG for NeuN, Cy3-conjugated donkey anti-rabbit IgG for GFAP, Iba1, FITC-conjugated donkey anti-rabbit IgG for PV, and Cy3-conjugated donkey anti-goat IgG for DCX were obtained from Jackson ImmunoResearch Inc., PA.

### Behavioral tests

Male mice, 8–10 weeks old were employed in the behavioral tests except for social behavior which employed 9–10-weeks-old female mice. Both genders were employed in the epilepsy tests, and 3-weeks-old mice were also used in the audiogenic epilepsy test. Behavioral tests were conducted based on previous protocols^25–34^ (Supplementary Methods). Fertility was monitored in 8-week-old mice. To examine parent-of-origin effects, paternal HT (HT-P) and maternal HT (HT-M) mice were generated by mating WT male with KO female and KO male with WT female, respectively. Animals from multiple litters were grouped and tested randomly and the experimenters were blinded to the genotype of the mice.

### Biochemical analysis and immunoassays

Tissues were collected from male mice aged 10–11 weeks old. Brain and liver tissues were homogenized in phosphate buffer saline (PBS) at pH 7.2 and subjected to MDA analysis by reaction with thiobarbituric acid (TBA)^35^. Blood sample was collected by cardiac excision from anesthetized mouse using a syringe pre-washed with 0.5% heparin in saline. After centrifuging the blood sample at 1500×*g* for 10 min at 4 °C, plasma was collected and subjected to reactive oxygen metabolite (ROM) analysis by reaction with *N*,*N*-dimethyl-*p*-phenylenediamine to quantitate *tert*-butylhydroperoxide (tBHP) equivalents^36^. ELISA kits for TNF-α and IL-6 (Invitrogen, USA) were used for brain assays according to manufacturer’s instructions.

### Immunohistochemical analysis

Male mice aged 10–11 weeks old were used. Cellular immunofluorescence was quantitated based on number of fluorescent cells or optical density using fluorescence-labeled antibodies^37^. Detailed methods are described in Supplementary Methods.

### Quantitative real-time PCR assays of mRNA expression

The mRNA expressions of various GABA_A_ receptor subunits in cerebrum and cerebellum tissues extracted from 10 to 11-weeks-old male mice were measured by real-time PCR (RT-PCR) using QuantiTect® reverse transcription kit (Qiagen) as described^38^. Briefly, total RNA was extracted from fresh brain tissue using Trizol reagent (Invitrogen Corp., CA) according to manufacturer’s instruction. Total RNA was converted to cDNAs using QuantiTect, and RT-PCR was performed using FastStart Universal SYBR Green Master (Roche) on the 7500 Real-Time PCR System (Thermo Fisher Scientific). *Actb* and *Pgk1* mRNAs were employed as reference. The primers employed in these RT-PCR experiments are described in Supplementary Table S1.

### Statistical analysis

Samples sizes were based on established practice and on our previous experience in respective assays^26,34^, and to some extent determined by breeding availability. The number of independent samples in each group is indicated by the individual points in the graphs, and also in the figure legends. Experimenters were blinded to animal genotype in the behavioral experiments and no sample was excluded from analysis. All data were presented as mean ± SEM. Statistical analysis was performed using Prism 5.0 (Graph Pad Software, La Jolla, CA, USA) and statistical significance was set at *p* < 0.05. Immunohistochemical data were analyzed by two-tailed unpaired *t*-test. All other data were analyzed using either one-way ANOVA followed by Newman–Keuls post-hoc test when one variable, i.e., genotype, was tested; or two-way ANOVA followed by Dunnett’s post-hoc test when two variables, i.e., genotype and drug dosage or genotype and gender, were tested. The statistical test used for each analysis is indicated in each figure legend and all data met the assumptions of the statistical tests. The variance between the statistically compared groups are similar.

## Results

### Compromised fertility in naive knockout mice

Naive KO mice displayed compromised fertility compared to WT mice, with *p* < 0.05 (Supplementary Table S2). Average litters of <4 were obtained when both parents were naive KO, as opposed to litters of more than 5 when naive male KO were mated with either naive female HT or WT. Normal litters of more than 6 were obtained for all other combinations of naive or non-naive parents.

### Reduced affective symptoms

**Fig. 1:**
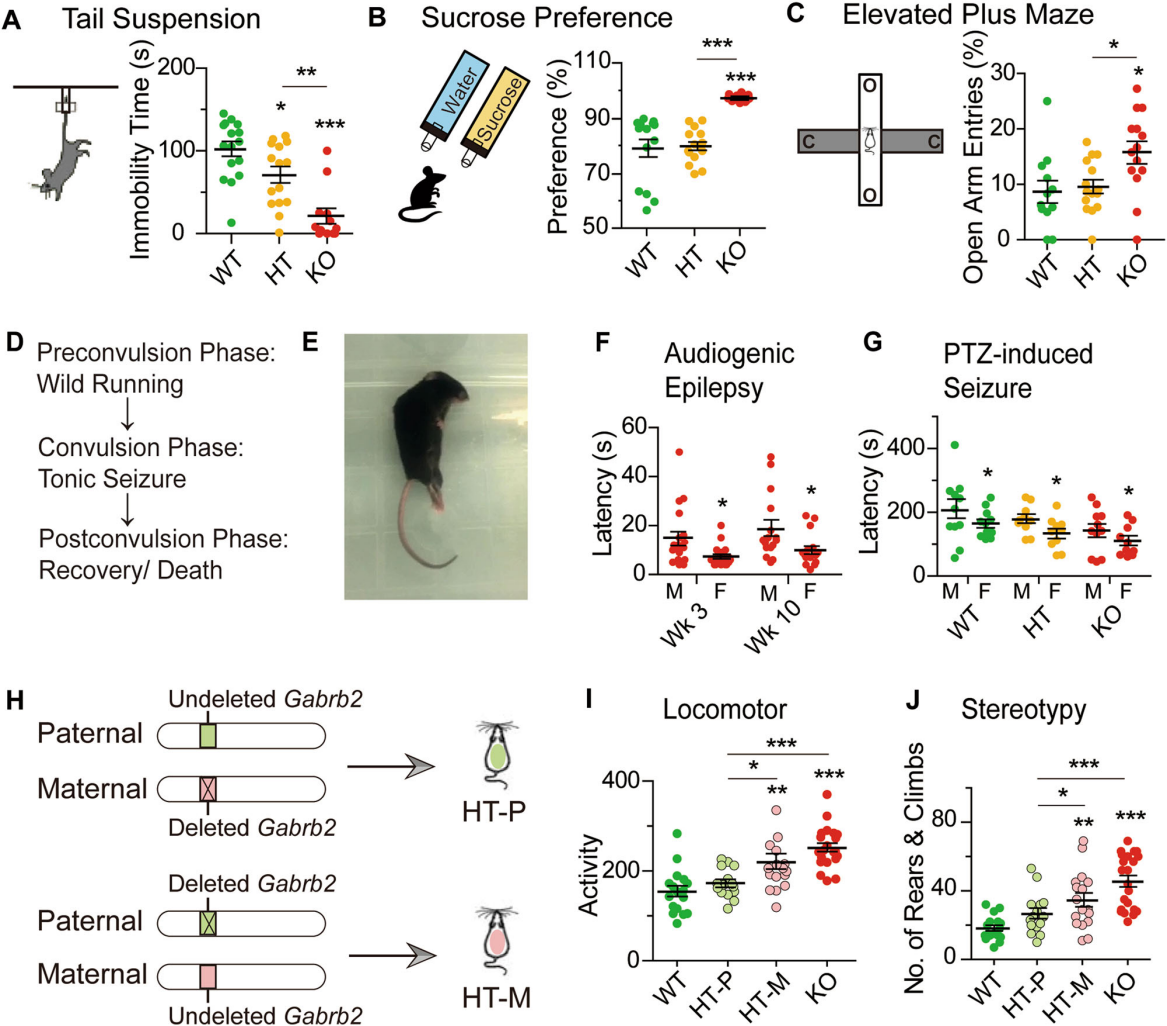
Epilepsy, affective symptoms, and behavioral genomic imprinting. **a** Tail-suspension test showing immobility time of mice suspended by the tail to a horizontal bar (WT male *n* = 16, HT male *n* = 15, KO male *n* = 12). **b** Sucrose-preference test showing preference in terms of sucrose solution consumed as percentage of total liquid consumption (WT male *n* = 14, HT male *n* = 15, KO male *n* = 12). **c** Elevated-plus maze test showing percentile entries into open arms (WT male *n* = 12, HT male *n* = 15, KO male *n* = 14). In schematic of (**c)**, O represents open arm and C represents closed arm. **d** Schematic of the different phases in audiogenic epilepsy. **e** KO mouse undergoing tonic seizure. **f** Audiogenic epilepsy: significant effect of gender on latency of audiogenic epilepsy was displayed by the mice (*F*_1,65_ = 12.12, *p* < 0.01; week-3 KO male *n* = 18, female *n* = 19; week-10 KO male *n* = 16, female *n* = 16). **g** PTZ-induced seizure: significant effects of gender (*F*_1,57_ = 6.12, *p* < 0.05) and genotype (*F*_2,57_ = 4.71, *p* < 0.05) on the latency of PTZ-induced seizure were displayed by the mice (WT male *n* = 11, female *n* = 11; HT male *n* = 10, female *n* = 10; KO male *n* = 11, female *n* = 10). In both parts (**f)** and **(g)**, male and female mice are labeled M and F, respectively; and data were analyzed using two-way ANOVA with Dunnett’s post-hoc test. **h** Schematic representation of two types of HT hemizygosity differing in parental origin of undeleted copy of *Gabrb2*. Upper: HT-P mouse displayed the phenotype of the *Gabrb2* of paternal origin (light green) while the maternal copy (pink) had been deleted. Lower: HT-M mouse displayed the phenotype of the *Gabrb2* of maternal origin (pink) while the paternal copy (light green) had been deleted. **i** Imprinting effect on locomotor activity (WT male *n* = 17, HT-P male *n* = 15, HT-M male *n* = 17, KO male *n* = 22). **j** Imprinting effect on behavioral stereotypy (WT male *n* = 17, HT-P male *n* = 15, HT-M male *n* = 17, KO male *n* = 22). Except for the epilepsy tests (**f**, **g**), statistical analysis was performed using one-way ANOVA with Newman–Keuls post-hoc test. WT mice are represented by green dots; HT by orange dots; HT-P by light green dots; HT-M by pink dots; and KO by red dots. Average *y* values ± SEM in the different plots are represented by horizontal bars. *p < 0.05, **p < 0.01, ***p < 0.001

When the depression and anxiety levels of HT and KO mice were assessed, the KO mice displayed significantly reduced immobility time in the tail-suspension test as well as increased sucrose preference compared to WT mice; whereas the HT mice displayed a smaller reduction in tail-suspension time and no significant increase in sucrose preference (Fig. 1a, b). Thus KO, and to a lesser extent HT, displayed a reduced level of depression relative to WT. In the elevated-plus maze test for anxiety, KO but not HT exhibited a significant increase of percentile entry into the open arms that was indicative of a decreased level of anxiety (Fig. 1c).

### Locomotor hyperactivity and behavioral stereotypy

**Fig. 2:**
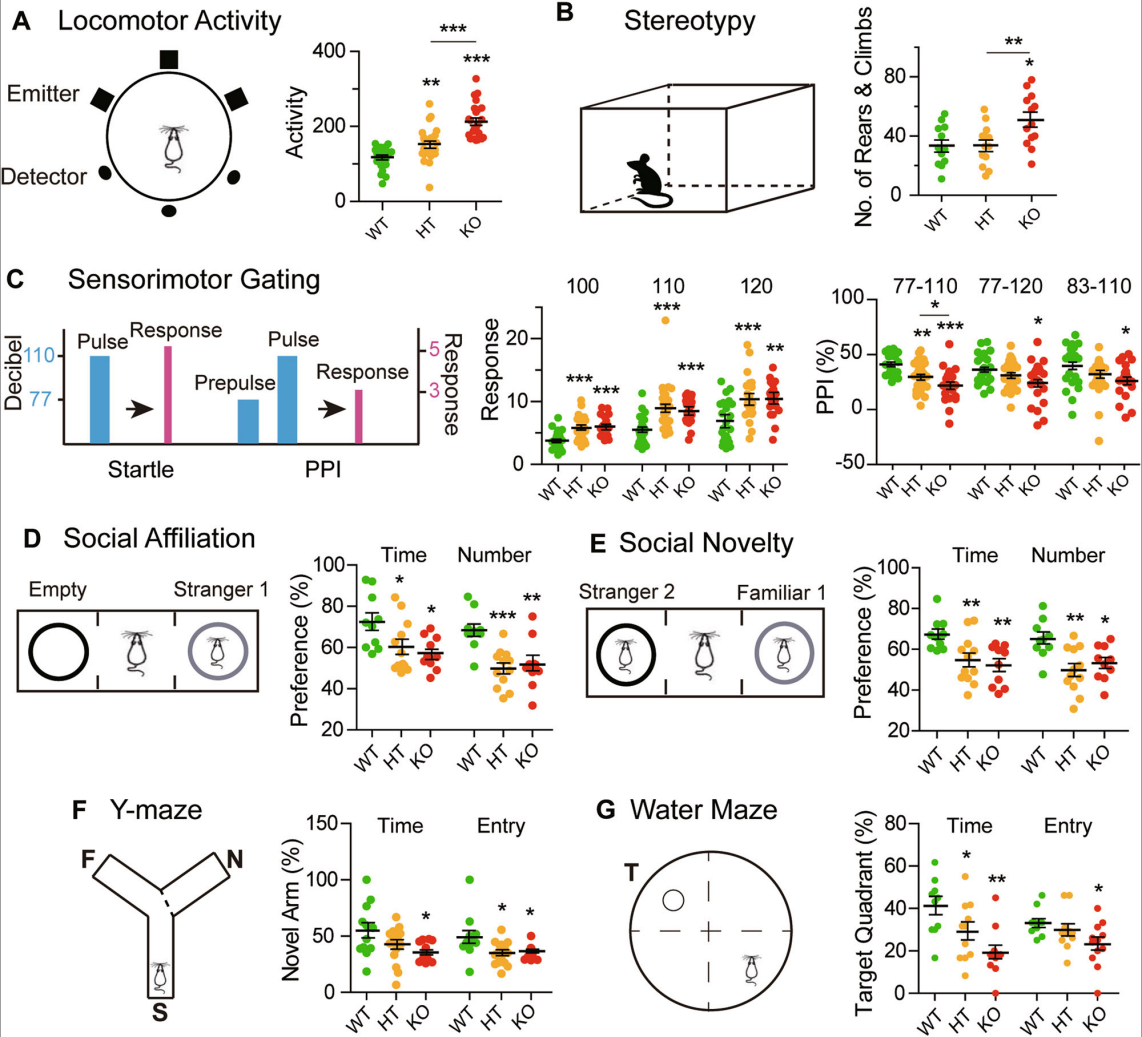
Schizophrenia-like behavior. **a** Locomotor activity: movement of mice across emitter beams during a 5-min interval (WT male *n* = 22, HT male *n* = 23, KO male *n* = 23). **b** Behavioral stereotypy: numbers of rears and climbs during a 5-min interval (WT male *n* = 12, HT male *n* = 13, KO male *n* = 13). **c** Sensorimotor gating based on PPI: acoustic startle response was shown for the 100, 110 and 120 dB pulse-alone trials; and PPI = 100% × [(Pulse-alone trial − Prepulse-pulse trial)/Pulse-alone trial] in the 77–110, 77–120, and 83–110 dB prepulse-pulse trials (WT male *n* = 21, HT male *n* = 25, KO male *n* = 21). Three-chamber social behavior tests for assessment of **d** social affiliation: preference for container holding Stranger-1 mouse relative to an empty container; and **e** preference for social novelty: preference for container holding Stranger-2 mouse relative to container holding Familiar-1 mouse. In both parts **(d**) and **(e**), assessment was performed based on time as well as number of visitations (WT female *n* = 10, HT female *n* = 12, KO female *n* = 10). **f** Y-maze test: percentile time spent in, or entries into, novel arm was monitored to measure spatial-working memory (WT male *n* = 12, HT male *n* = 15, KO male *n* = 14). S represents the start-arm in the schematic, F the familiar arm, and N the novel arm. **g** Morris water maze: percentile time spent in, or entries into, the target quadrant was monitored to measure spatial-reference memory (WT male *n* = 10, HT male *n* = 11, KO male *n* = 12). T in the schematic represents target quadrant that had housed the submerged platform. Statistical analysis was performed using one-way ANOVA with Newman–Keuls post-hoc test. WT is represented by green dots; HT by orange dots; and KO by red dots. Average *y* values ± SEM in the different plots are represented by horizontal bars. *p < 0.05, **p < 0.01, ***p < 0.001

When psychotic agitation was assessed based on locomotor hyperactivity and behavioral stereotypy, both KO and HT mice displayed locomotor hyperactivity compared to WT, with KO being more hyperactive than HT, showing that *Gabrb2* gene dosage was negatively correlated with locomotor activity (Fig. 2a). Regarding behavioral stereotypy, more rearing and climbing were observed in KO compared to both WT and HT, whereas there was no significant difference between WT and HT, again demonstrating the presence of gene dosage effects (Fig. 2b). There was no significant difference between KO, HT, and WT in either circling or sniffing stereotypy (Supplementary Fig. S1a, b).

### Sensorimotor gating deficit

In the PPI test for core schizophrenia-like symptoms relating to sensorimotor gating, the acoustic startle response was significantly larger in HT and KO mice than in WT mice for 100, 110, and 120 dB sounds, suggesting that both HT and KO were more sensitive to acoustic stimulus than WT. Significant decreases in PPI were observed in KO relative to WT for the 77–110, 77–120, and 83–110 dB prepulse-pulse combinations, in HT for only the 77–110 dB combination (Fig. 2c), and in neither KO nor HT for the 83–120 dB combination (Supplementary Fig. S1c), indicating that reduced copies of *Gabrb2* brought about sensorimotor gating deficits resembling those observed in schizophrenia.

### Deficits in social functions

In the three-chamber test, WT displayed greater social affiliation and preference for social novelty compared to HT and KO based on either time or number of close contact visitations, thereby establishing deficits of HT and KO with respect to both social affiliation and preference for social novelty (Fig. 2d, e). Since there was no significant difference between HT and KO regarding either time or number of visitations when they were compared against one another, no gene dosage effect was evident.

### Impairment of cognitive functions

For hippocampus-dependent memory functions, the Y-maze measures spatial-working memory, and the Morris water maze measures spatial-reference memory^39^. In the Y-maze test, reduced novel-arm visitation time and entries were displayed by KO mice, but only reduced entries were displayed by HT mice (Fig. 2f). In the Morris water maze test, both reduced target-quadrant visitation time and entries were displayed by KO mice, but only reduced visitation time was displayed by HT mice (Fig. 2g). Therefore, the KO and HT mice were inflicted with deficits in both spatial-working memory and spatial-reference memory, with significant gene dosage effect.

### Audiogenic epilepsy and chemical-induced seizure with gender effect

When exposed to white noise, 95% or more of both female and male KO mice, but none of the HT and WT mice, were susceptible to audiogenic epilepsy characterized by wild running followed by tonic seizure (Fig. 1d–f, Supplementary Video, Supplementary Table S3); whereas PTZ induced seizure in WT, HT as well as KO mice (Fig. 1g). For audiogenic seizure, the average latency to seizure was shorter for female KO than for male KO at both week-3 and week-10; and both KO and HT were more susceptible than WT toward PTZ-induced seizure with the females again displaying a shorter latency than the males in each of the WT, HT, and KO groups (Fig. 1f, g). The susceptibility of KO mice to audiogenic epilepsy was in accord with the findings of comorbidity between schizophrenia and epilepsy^40,41^.

### Parent-of-origin effects

Parent-of-origin effects were examined by dividing HT mice into the paternal HT (HT-P) and maternal HT (HT-M) groups, with acquisition of a normal copy of *Gabrb2* from father or mother respectively (Fig. 1h). HT-M mice exhibited lower levels of locomotor hyperactivity and stereotypy of repetitive climbing and rearing than KO mice but higher than both HT-P and WT mice in theses regards, whereas there was no significant difference between HT-P and WT mice (Fig. 1i, j). On the other hand, with respect to the level of depression assessed by the tail-suspension test, both HT-P and HT-M exhibited significantly shorter immobility time than WT and longer immobility time than KO, with no significant difference between HT-P and HT-M (Supplementary Fig. S1d).

### Reversal of phenotypic alterations by risperidone and diazepam

**Fig. 3:**
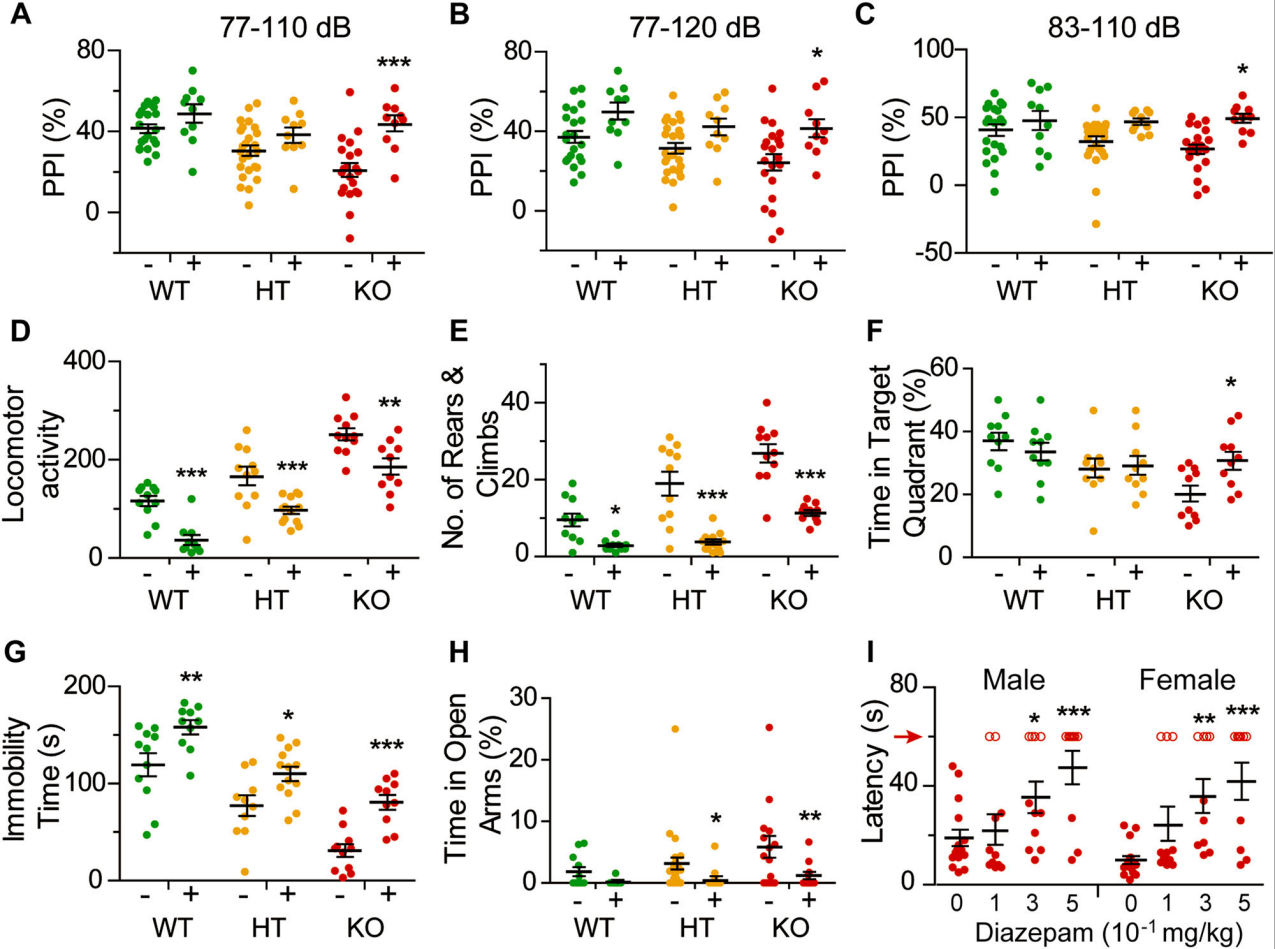
Reversal of KO behavioral phenotypes by risperidone and diazepam. The behavior of WT, HT, or KO mice administered with 0.3 mg/kg risperidone i.p was compared that of control mice administered with saline in: **a**–**c** PPI trials with significant effects of risperidone in the 77–110, 77–120, and 83–110 dB prepulse-pulse trials (*F*_1,92_ = 19.20, *p* < 0.001; *F*_1,92_ = 16.80, *p* < 0.001; and *F*_1,92_ = 15.71, *p* < 0.001, respectively), and significant effects of genotype in the 77–110 and 77–120 dB prepulse-pulse trials (*F*_2,92_ = 8.04, *p* < 0.001; and *F*_2,92_ = 3.53, *p* < 0.05, respectively). Saline group: WT male *n* = 21, HT male *n* = 25, KO male *n* = 21; risperidone group: WT male *n* = 10, HT male *n* = 10, KO male *n* = 10. **d** Locomotor activity test with significant effects of risperidone (*F*_1,95_ = 49.87, *p* < 0.0001) and of genotype (*F*_2,95_ = 51.78, *p* < 0.0001). Saline group: WT male *n* = 11, HT male *n* = 11, KO male *n* = 11; risperidone group: WT male *n* = 10, HT male *n* = 13, KO male *n* = 10. **e** Behavioral stereotypy test based on rears and climbs with significant effects of risperidone (*F*_1,60_ = 72.57, *p* < 0.0001) and of genotype (*F*_2,60_ = 25.33, *p* < 0.0001). Saline group: WT male *n* = 11, HT male *n* = 11, KO male *n* = 11; risperidone group: WT male *n* = 10, HT male *n* = 13, KO male *n* = 10. **f** Morris water maze test with significant effect of genotype (*F*_2,54_ = 6.20, *p* < 0.01). Saline group: WT male *n* = 10, HT male *n* = 10, KO male *n* = 10; risperidone group: WT male *n* = 10, HT male *n* = 10, KO male *n* = 10. **g** Tail-suspension test with significant effects of risperidone (*F*_1,59_ = 32.92, *p* < 0.0001) and of genotype (*F*_2,59_ = 43.82, *p* < 0.0001). Saline group: WT male *n* = 11, HT male *n* = 10, KO male *n* = 11; risperidone group: WT male *n* = 10, HT male *n* = 13, KO male *n* = 10. **h** Elevated-plus maze test with significant effect of risperidone (*F*_1,96_ = 13.29, *p* < 0.001). Saline group: WT male *n* = 12, HT male *n* = 25, KO male *n* = 16; risperidone group: WT male *n* = 12, HT male *n* = 25, KO male *n* = 12. **i** Audiogenic epilepsy with significant effects of diazepam (*F*_3,87_ = 12.47, *p* < 0.001). Saline group: KO male *n* = 16, KO female *n* = 16; 0.1 mg/kg diazepam group: KO male *n* = 11, KO female *n* = 11; 0.3 mg/kg diazepam group: KO male *n* = 11, KO female *n* = 10; 0.5 mg/kg diazepam group: KO male *n* = 10, KO female *n* = 10. Latency periods were capped at the maximum cutoff of 60 seconds. In **(a**–**h)**, ‘+’ denotes animals administered with 0.3 mg/kg risperidone i.p, and ‘−’ animals administered with saline i.p. In (**i)**, the different groups were treated with 0–0.5 mg/kg diazepam i.p. WT is represented by green dots, HT by orange dots, and KO by red dots except in (**i)** where KO mice reaching the 60-s cutoff are represented by red open circles. Average *y* values ± SEM in the different plots are represented by horizontal bars. Statistical analysis was performed using two-way ANOVA with Dunnett’s post-hoc test; *p < 0.05, **p < 0.01, and ***p < 0.001 in all the post-hoc tests

The antipsychotic drug risperidone has been employed for the clinical treatment of schizophrenia^42^. Administration of 0.3 mg/kg risperidone i.p. significantly reversed the PPI inhibition to levels similar to or even higher than those of untreated WT (Fig. 3a–c). Locomotor activity was suppressed in WT, HT and KO; the suppression in HT, but not that in KO, sufficed to bring the activity level down to that of untreated WT (Fig. 3d). The increased numbers of stereotypic rears and climbs in both HT and KO, however, could be rolled back completely (Fig. 3e). There was a small reversal of the cognitive defect of KO by the drug in the Morris water maze (Fig. 3f), but no significant effect on the Y-maze test or social behavior tests (Supplementary Fig. S2). The drug prolonged the immobility time in the WT, HT, and KO mice in the tail-suspension test (Fig. 3g), and reduced the percentage time spent by HT and KO mice in the open arms on the elevated-plus maze (Fig. 3h), indicating its effectiveness in partially reversing the decreased level of depression, and fully reversing the decreased level of anxiety in HT and KO mice.

Upon administration of 0.3 or 0.5 mg/kg i.p. of the GABA_A_ receptor agonist diazepam to the KO mice, the latency to audiogenic epilepsy was increased in both male and female KO mice (Fig. 3i), and the prevalence of audiogenic epilepsy was reduced (Supplementary Table S3). However, 0.5 mg/kg diazepam i.p. also induced sedation based on the holeboard test (Supplementary Fig. S3).

### Immunohistochemical and biochemical alterations in the brain

**Fig. 4:**
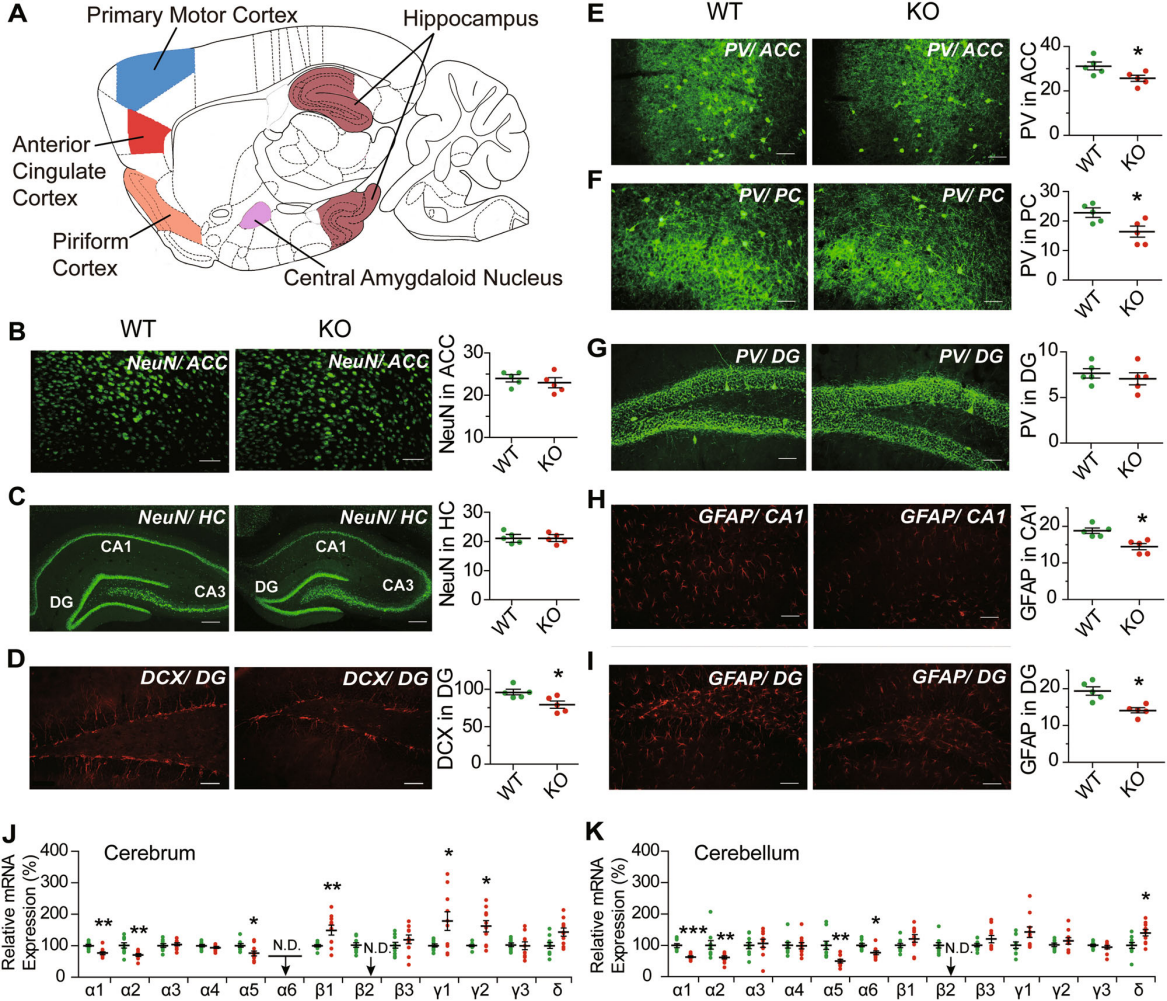
Neuron and astrocyte immunohistochemical analysis and mRNA expression of GABA_A_ receptor subunits. **a** Outline of sagittal section of mouse brain, showing the brain regions analyzed using immunohistochemical staining. **b**–**i** Coronal sections stained through fluorescence-labeling of: **b** NeuN in anterior cingulate cortex (ACC); **c** NeuN in hippocampus (HC); **d** DCX in dentate gyrus (DG); **e** parvalbumin (PV) in ACC; **f** PV in piriform cortex (PC); **g** PV in DG; **h** GFAP in CA1 region; and **i** GFAP in DG. The DG, CA1, and CA3 regions in hippocampus are indicated in part (**c**). NeuN and PV immunofluorescence are displayed in green (scale bar = 300 μm), and that of DCX and GFAP and DCX displayed in red (scale bar = 120 μm). The plots show immunofluorescence densities for WT (green dots) and KO (red dots) estimated in terms of average area optical density (in parts **b**, **c**, **e**, **h**, and **i**), or in terms of the number of immunoreactive cells (in parts **d**, **f**, **g**); in each instance, five randomly selected images from each of five WT or KO male mice were examined. The levels of mRNA expression for 13 different GABA_A_ receptor subunits in WT and KO mouse cerebrum (**j**) and cerebellum (**k**) were measured using quantitative RT-PCR, and the mRNA levels in KO mice were normalized to the average expression level in WT (*n* = 10 in each group). The measured expression levels in WT, KO as well as HT brains are given in Supplementary Table S4. Statistical analysis was performed using unpaired *t*-test for immunohistochemical staining and one-way ANOVA with Newman–Keuls post-hoc test for mRNA quantitation. Average *y* values ± SEM in the different plots are represented by horizontal bars; WT is represented by green dots and KO by red dots. N.D. represents non-detectable; *p < 0.05, **p < 0.01, ***p < 0.001

**Fig. 5:**
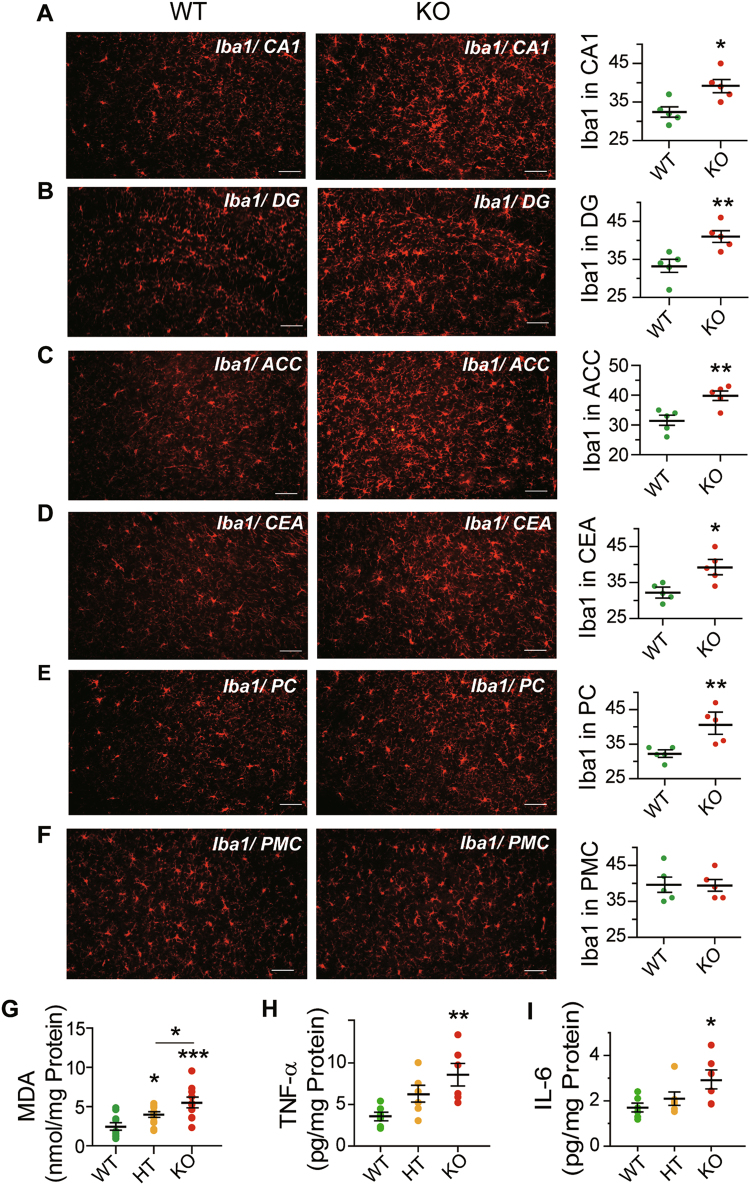
Neuroinflammation in *Gabrb2* KO mice. Coronal sections were stained through fluorescence-labeling of Iba1 on microglia in: **a** CA1 of hippocampus; **b** dentate gyrus (DG); **c** anterior cingulate cortex (ACC); **d** central amygdaloid nucleus (CEA); **e** piriform cortex (PC); and **f** primary motor cortex (PMC). In **(a**–**f)**, the number of Iba1-positive cells in WT or KO was estimated from five randomly selected images for each of five WT or KO males. Brain levels of **g** MDA (WT male *n* = 10, HT male *n* = 12, KO male *n* = 10), **h** TNF-α (WT, HT, and KO males, *n* = 6 in each group) and **i** IL-6 (WT, HT, and KO males, *n* = 6 in each group) were measured and expressed per mg protein of sample analyzed. Statistical analysis was performed using unpaired *t*-test for immunohistochemical measurements and one-way ANOVA with Newman–Keuls post-hoc test for the biochemical measurements; WT is represented by green dots, HT by orange dots, and KO by red dots. Average *y* values ± SEM in the different plots are represented by horizontal bars; *p < 0.05, **p < 0.01, ***p < 0.001

There was no extensive change in the optical density (O.D.) of neuronal immunostaining obtained with fluorescent-labeled anti-NeuN between KO and WT mice in the anterior cingulate cortex (ACC) or hippocampus (HC), or the number of PV-positive neurons in the dentate gyrus (DG) in hippocampus (Fig. 4a–c, g). In contrast, the O.D. of PV-staining in neurons in the ACC, and the number of PV-positive neurons in the piriform cortex (PC) were decreased in KO mice relative to WT mice (Fig. 4e, f). The number of DCX-positive newborn neurons in DG, and the O.D. of GFAP-positive astrocytes in CA1 and DG of hippocampus were all decreased in KO compared to WT (Fig. 4d, h, i). There was no significant change in the O.D. of GFAP-positive astrocytes in ACC, or the number of PV-positive neurons in CA1 in hippocampus or central amygdaloid nucleus (CEA) in KO compared to WT (Supplementary Fig. S4). On the other hand, the number of Iba1-positive microglia was increased in KO compared to WT mice in CA1, DG, ACC, CEA, and PC, but not in the primary motor cortex (PMC) (Fig. 5a–f).

The brain levels of oxidative stress measured by MDA were elevated in HT and KO compared to WT mice, with significant dosage effect between HT and KO (Fig. 5g), but neither in liver nor blood as measured by ROM (Supplementary Fig. S5). The brain levels of the inflammatory cytokines TNF-α and IL-6 were elevated in KO, but not in HT, compared to WT mice (Fig. 5h, i).

### Differential expression of GABA_A_ receptor subunits

When *Gabrb2* exon 7-specific primers were employed to quantitate *Gabrb2* expression based on its mRNA level, expression was found to be reduced in HT mice compared to WT, and non-detectable (N.D.) in KO, in both cerebrum and cerebellum. The mRNA levels of different subunits of GABA_A_ receptors in the cerebrum or cerebellum in KO (red dots) compared to WT (green dots) showed significant upregulations for *Gabrb1*, *Gabrg1*, and *Gabrg2*, but downregulations for *Gabra1*, *Gabra2*, and *Gabra5* in the KO cerebrum; in contrast, there were significant upregulation for *Gabrd*, but downregulations for *Gabra1*, *Gabra2*, *Gabra5* and *Gabra6* in the KO cerebellum (Fig. 5j, k, Supplementary Table S3). A factor underlying the various downregulations was likely the loss of more than 50% of total GABA_A_ receptors in the KO mice^21^, and the various upregulations could be indicative of replacement of the missing β_2_ subunit in GABA_A_ receptors by the upregulated subunits.

## Discussion

Previously, analysis of schizophrenia genomics pointed to *GABRB2* as a key susceptibility gene for schizophrenia^7–13^. Alternative splicing of the gene transcript gave rise to two major products comprising an ancient short β_2_ isoform and a derived, positively selected long β_2_ isoform, and only the long isoform carries a potential phosphorylation site at Thr^365^. GABA_A_ receptors bearing the long β_2_ isoform were fatigued and underwent current rundown more rapidly that those with the short isoform upon repeated stimulation in the presence of low ATP concentration, thereby diminishing the strength of inhibitory signaling of GABAergic neurons. This effective linkage of neuroinhibition to energy supply made possible by the long isoform was positively selected in evolution, for it would disinhibit actions related to striving, hunting, and pursuit when food is limited. There were reductions of 21.7% in long-isoform expression, 13.4% in short isoform expression and 15.8% in their combined expression in schizophrenic brain, resulting in twin deficits of total β_2_ subunit and the long isoform relative to short isoform^12^.

### Pathophysiological mechanisms of *Gabrb2* knockout

In agreement with schizophrenia genomics, Figs. 1 to 5 show that the *Gabrb2* KO mice exhibited behavioral and cognitive changes similar to those observed in schizophrenia, including: neuroinflammation with increased oxidative stress^43^, increased pro-inflammatory cytokines^44^, and microglial activation^45–47^, as well as the comorbidities of hyperactivity^48^, decreases in cell proliferation in hippocampus^4,49,50^, and epilepsy^40,41^.

Based on immunostaining, the complete loss of *Gabrb2* affected four types of cells in the brain prominently: viz. decreases in DCX-positive newborn neurons, PV-positive neurons and GFAP-positive astrocytes, together with widespread increases in Iba1-positive microglia (Fig. 4b–i, Fig. 5a–e). Since PV-positive GABAergic interneurons are important to gamma-band synchrony and cognition^51,52^, their decreases could contribute to the deficits in cognition revealed by the Y-maze and water maze tests (Fig. 2f, g). The increases in Iba1-positive microglia together with increased brain levels of oxidative stress and the pro-inflammatory cytokines TNF-α and IL-6 (Fig. 5g–i) pointed to the presence of regional neuroinflammation. Notably, the decreases of newborn neurons and astrocytes along with microglia activation in the hippocampus underlined the importance of hippocampus in the phenotypes of *Gabrb2*-knockout mice (Fig. 2f, g), and confirmed earlier suggestions of hippocampal involvement in schizophrenia^4,5^.

Unlike classical phasic GABA_A_ receptor-mediated inhibition, tonic GABA_A_ receptor-mediated inhibition results from the activation of extrasynaptic receptors by low concentrations of ambient GABA in the extracellular space^53^, and tripartite synapses formed by presynaptic neuron, postsynaptic neuron and astrocyte enable bidirectional communication between astrocytes and neurons^54^. Moreover, the GABAergic astrocytes modulate the GABAreceptive microglia, and GABA suppresses the reactive response of both astrocytes and microglia to inflammatory stimulants, leading to a reduced release of the inflammatory cytokines IL-6 and TNF-α^55,56^. Evidence also suggests the participation of neuron–astrocyte–microglia triad in the regulation of neuroinflammation in hippocampus^57^. Accordingly, the observed decreases in newborn neurons, PV-positive interneurons and astrocytes, and increases in microglia (Figs. 4 and 5) may be expected to alter significantly the interactions between interneurons, astrocytes and microglia, giving rise to regional neuroinflammation and development of schizophrenia-like and comorbid phenotypes in the *Gabrb2*-knockout mice.

A substantial portion of schizophrenia cases experience episodes of mood disorder as well as periods of non-affective psychosis, and the distinction between schizophrenia and schizoaffective disorder is marked with some difficulty^58^. The decreased depression-like behavior in the tail-suspension and sucrose-preference tests, and anxiety-like behavior in the elevated-plus maze in *Gabrb2*-knockout mice (Fig. 1a–c), indicated a separation of the schizophrenia type symptoms from comorbid affective symptoms. Insofar that anxiety could be regulated through extrasynaptic inhibition^59^, the alterations in GABAergic interneurons and tripartite synapses brought about by *Gabrb2* knockout could also be factors in the decreases of depression- and anxiety-like symptoms.

### Comparison of animal models for schizophrenia

A variety of animal models have produced limited phenotypic resemblances with schizophrenia and its comorbidities, as in the case of *Disc-1*, *Nrg1*, and *ErbB4* knockouts; and in some instances included also phenotypic divergences, as in the case of unimpaired hippocampal-dependent memory and lack of social-interaction reduction in amphetamine models, lack of sustained PPI deficit in phencyclidine models, increased PPI in dysbindin knockout, and reduced locomotion in reelin knockout^60^. Likewise, there were lack of social-interaction deficit in dopamine-transporter knockout^61^; decreased locomotor activity, and lack of significant change in PPI or learning and memory in *Gabra1* knockout^62^; and improved performance in the water maze test in *Gabra5* knockout^63^. Mice hypomorphic in the *N*-methyl-d-aspartate (NMDA) R1 subunit (NR1) exhibited decreased PPI and social affiliation^64^, but the induction of schizophrenia-like symptoms by partial ablation of NR1 from GABAergic neurons, a majority of which were parvalbumin-positive, pointed to the mediation of schizophrenia-like phenotypes by NMDA-receptors on GABAergic neurons^65^, in which case the schizophrenia-like phenotypes could stem from disturbance of either the NMDA-receptors or GABA_A_-transmission or both. In contrast, in *Gabrb2* knockout, the absence of the GABA_A_-receptor β_2_ subunit was clearly the root of the schizophrenia-like phenotypes and comorbidities of the KO mice, with no major divergence from the positive symptoms, negative symptoms and cognitive deficit of schizophrenia, and reversible in part or in full by risperidone for a number of the symptoms. Furthermore, the dissimilarities between the symptoms of schizophrenia and those observed so far in the *Gabra1* and *Gabra5* knockouts suggest that the extensive similarities between *Gabrb2* knockout and schizophrenia are not readily shared by knockouts of other subunits of GABA_A_ receptors.

### *GABRB2*-origin of schizophrenia

Earlier findings on schizophrenia genomics, gene expression and alternate-splicing of the β_2_ receptor subunit have pointed to a key role played by *GABRB2* genotypes and haplotypes in the disease. However, because schizophrenia and its comorbidities are associated with such a variety of symptoms, it is difficult to determine the minimum number of genetically perturbed genes required for disease initiation. In this regard, the *Gabrb2*-knockout model demonstrated that deletion of *Gabrb2* alone was sufficient cause for a range of the schizophrenia-like positive symptoms, negative symptoms and cognitive impairments in the homozygous KO mice, thereby enabling the proposal of a *GABRB2*-origin theory of schizophrenia. That the hemizygous HT mice were symptomatic indicated clearly just how low is the inhibitory-power redundancy in some of the β_2_-containing GABAergic receptors in the brain. The twin reductions in total β_2_ expression and long-to-short β_2_ isoform ratio induced by the schizophrenia susceptibility-enhancing genotypes and haplotypes in the AluYi6AH-151 region of *GABRB2* would thus lead to inadequate inhibitory power on account of β_2_ shortage and diminished long-to-short isoform ratio, and therefore illness.

To trace the etiological pathway from a shortage of β_2_ subunit, especially its long isoform, it may be noted that neural stability depends on a balance between excitatory neurotransmitters such as glutamate, and inhibitory ones comprising mainly GABA. In the face of a shortage of any GABA_A_ subunit, risk of disinhibition would be enhanced, particularly in brain regions such as hippocampus where the neurons are ~90% glutamatergic pyramidal cells, and only 10% GABAergic non-pyramidal cells^66^. The non-lethality of two-copy *Gabrb2* knockout in mice^21^ show that the brain can extensively maintain function by replacing the β_2_ subunit in GABA_A_ receptors with other subunits, and the subunit upregulations in KO mice suggest that, upon deletion of *Gabrb2*, the β_2_ subunit in GABA_A_ receptors could be replaced by β_1_, γ_1_, and γ_2_ in the cerebrum, or by δ in the cerebellum (Fig. 4j, k). Since there are β_1_ and γ_2_ but limited δ and little γ_1_ in the hippocampus available for β_2_ replacement^67^, insufficient or unsatisfactory replacement of β_2_ by other subunits in some hippocampal GABA_A_ receptors in the event of a β_2_ deficit could represent a significant factor of defective hippocampal function in schizophrenia or in the *Gabrb2* KO mice.

With disinhibition, overstimulation of neurons by glutamate leads to influx of calcium, excitotoxicity, and cell death^68^, producing cell debris. Microglia activation is inducible by pathogens or cell debris, and the occurrence of microglial activation in various brain regions but not the primary motor cortex (Fig. 5a–f) indicated that microglial activation in the KO mouse brain was regional and induced by cell debris rather than infection. Microglial activation and neuroinflammation could in turn give rise to the symptoms and comorbidities of schizophrenia^45–47^. The immune-response nature of microglial activation is consistent with the association of schizophrenia with the major histocompatibility complex (MHC)^19,69^. In severe traumatic brain injury, excitotoxicity gives rise to posttraumatic epilepsy in 20% of cases, and 50% in cases with penetrating head wounds^70^, indicating that the audiogenic epilepsy of the *Gabrb2* KO mice could likewise be the consequence of excitotoxicity. The observation of audiogenic epilepsy in only KO but not HT mice suggests that a deeper GABAergic deficit than that inflicted by *Gabrb2* hemizygosity would be required for epileptogenesis.

In conclusion, the advantage of the *GABRB2*-origin theory of schizophrenia resides in the multiplicity of its supportive evidence: (a) The SNPs in the vicinity of the AluYi6AH-151 insertion of *GABRB2* were correlated with schizophrenia with odds ratios of 1.93–2.50, and also with both total β_2_ subunit and its long-to-short isoform ratio, indicating that their associations with schizophrenia arose directly from their regulation of β_2_ expression and alternative splicing^7–15^. The evidence for their positive selection indicates their functional importance, haplotype analysis points to haplotypes H26 and H73 as protective, and H19 and H81 as risk-conferring toward schizophrenia^11^, and the correlations between genotypes and antipsychotics dosage among schizophrenia patients^16,18^ confirm the clinical relevance of these SNPs. (b) The more rapid attenuation of long β_2_ isoform-containing GABA_A_ receptors compared to short isoform-containing ones in the presence of low ATP indicates that the long-to-short isoform ratio correlated to schizophrenia pertains to a clearly defined electrophyiological characteristic of β_2_-containing GABA_A_ receptors. (c) *Gabrb2* knockout in mice produced a range of schizophrenia-like symptoms and comorbidities, some of which are reversed partly or fully by the antipsychotic risperidone. (d) The theory can account for regional microglial activation in the brain of KO mice based on the varied GABA_A_ receptor subunit compositions in different brain regions leading to unsatisfactory replacement of β_2_ by other subunits in regions such as hippocampus and anterior cingulate cortex but not the primary motor cortex. (e) The theory can account for the otherwise difficult to explain complete recovery achieved in 25% of schizophrenia cases^71^ based on the ~35% increases in total β_2_-subunit expression and long-to-short β_2_ isoform ratio in human brains between the ages of 30 and 50, which would ameliorate by middle age the disease-eliciting shortages of β_2_ subunit and its long isoform in adolescents and young adults^15^; and the marked differences in the compositions and presumably properties of GABA_A_ receptors in early postnatal compared to adult rat brain^72^ may furnish a possible basis for the much lower incidence of schizophrenia in young children compared to adolescents. (f) The audiogenic epilepsy of KO mice helps to explain the comorbidity of epilepsy with schizophrenia. (g) The pervasive connections of disease-prone β_2_-containing GABAergic interneurons in the brain could facilitate coalescence of the relatively minor defects of a large array of genes into a seriously debilitating multigene disease, thereby explaining the remarkably wide spectrum of schizophrenia phenotypes and comorbidities, as well as the finding of numerous schizophrenia SNPs with low odds ratios typically around 1.10 and rarely exceeding 1.20, denoting only a very small effect on disease risk^73^.

Based on the *GABRB2*-origin of schizophrenia, functionally defective β_2_ subunit-containing GABA_A_ receptors would begin the etiological changes, and proceed to involve wide ranging neuroreceptor systems and brain structures to produce the spectrum of symptoms and comorbidities characteristic of schizophrenia. This is evident from the possible modulation of PPI, which has been regarded as an endophenotype of schizophrenia, by interventions at the dopamine, NMDA, 5HT_2C_, CB1 cannabinoid, neurotensin-1, adenosine A(2A), alpha-7 nicotinic and histamine H3 receptors, revealing the participation of these diverse receptor systems in shaping a single disease phenotype^74^. On account of such networking between receptor systems, a *GABRB2*-origin of schizophrenia is entirely compatible with drug development against the disease targeting widely at the dopamine, glutamate, GABA, acetylcholine, serotonin, and hormonal systems, necessitated particularly by the shortfall of effective medications for the negative symptoms and cognitive deficits^75,76^. As illustrated in the present study, risperidone could moderate a number of the *Gabrb2*-knockout-induced phenotypes even though the drug is known to act on catecholamine receptors (mainly dopamine 2, and alpha 1 & 2 adrenoceptors) and 5HT2 receptors^42^. Consequently, a deeper understanding of how different neurotransmitter and neuronal systems interact in schizophrenia to generate its symptoms and comorbidities, obtained through integrated approaches including clinical and genetic studies, investigation of postmortem schizophrenic brains and thorough analysis of the *Gabrb2*-knockout model, will be important for not only delineation of the complex etiology of schizophrenia, but also expedited searches for improved drugs to treat the disease.

## Electronic supplementary material

Supplementary Materials

Supplementary Video
