# Supplementary material for: Gabrb2-knockout mice displayed schizophrenia-like and comorbid phenotypes with interneuron–astrocyte–microglia dysregulation
Source: Transl Psychiatry. 2018 Jul 17;8:128. doi: 10.1038/s41398-018-0176-9 (PMC6048160; doi:10.1038/s41398-018-0176-9)
Supplement: Supplementary file 1 — Supplementary Materials [file 41398_2018_176_MOESM1_ESM.docx]

**SUPPLEMENTARY METHODS**

**Mouse genotyping**

Genotyping was carried out on 3-week old mice according to the protocol provided by Taconic Farms, Inc. (New York). Briefly, DNA from ear tag was extracted following alkaline lysis, and subjected to PCR using either of two primer pairs: one specific for the neomycin gene (*Neo*) and the other specific for exon 7 of *Gabrb2* (Supplementary Table S1) with the resulting products having molecular weights of 375 bp and 153 bp respectively, which were separated and visualized using agarose gel electrophoresis. Genotype was determined by the presence of *Gabrb2* only (WT), both *Gabrb2* and *Neo* (HT) or *Neo* only (KO). Knockout of *Gabrb2* in KO mice was further confirmed by non-detection of exon-1 mRNA expression by RT-PCR using exon 1-specific primers (Supplementary Table S1).

**Animal behavior tests**

*Prepulse inhibition (PPI) test*

Standard startle chambers (San Diego Instruments, CA) were employed, and PPI test was performed basically as described^1^. The mice were allowed 2-min acclimatization before undergoing a series of prepulse-pulse trials of 77-110, 83-110, 77-120 and 83-120 dB with white noise background. Pulse-alone trials were conducted to examine genotype-dependent differences in hearing ability and startle response. Each PPI test consisted of a complete set of pulse and prepulse-pulse trials performed randomly and lasted for 35 min. Percentage PPI for each trial was given by %PPI = [(Pulse alone trial – Prepulse-pulse trial)/ Pulse-alone trial] x 100.

*Locomotor activity test*

The ZIL-2 apparatus (Beijing Institute of Materia Medica, China) employed for recording locomotor activity^2^, was 60cm x 60cm x 12cm in dimensions, and consisted of four circular plastic boxes each equipped with six evenly spaced infrared beam-photocell setups for recording movement over a 5-min test period.

*Behavior stereotypy tests*

The mouse was first habituated in an empty cage for 5 minutes before behavioral stereotypy was assessed by measuring the frequency of circling, self-sniffing or climbing-and-rearing behavior over a 5-min test period^3^.

*Y-maze test*

The Y-maze test apparatus consisted of 3 arms positioned at an angle of 120° from each other^4^. Prior to the test, the novel arm was closed and exploration time of 2 min was allowed for the mouse to familiarize with the apparatus. After a 30-min interval, the mouse was reintroduced into the apparatus with the novel arm now opened. The number of entries and the time spent in the novel arm were recorded for 5 min, and their ratios over total numbers of arm entries and total time spent in all the arms were recorded. Arm entry was scored when all four paws were placed within the arm.

*Morris water maze (MWM) test*

The water maze consisted of a circular pool (160 cm in diameter) filled with opaque water (30 cm depth) at 25±1°C. There was a submerged escape platform (11 cm in diameter) at 1 cm below water surface in the middle of one of the quadrants^5^. The behavior of the animal was monitored with a video camera mounted above the pool. In the training trials from day-1 to day-4, each day the mouse was subjected to four 60 s trials starting at four different positions with a 30 min interval in between staying in its home cage. Each trial began with the mouse in the pool facing the sidewalls. If the mouse failed to locate the platform within 60 s, it was guided to the platform by the experimenter. Otherwise, when the mouse climbed onto the platform, it was allowed to stay on the platform for 10 s before being returned to its home cage. The submerged platform was removed on day-5, and memory test was conducted in a 60 s test trial, during which the time spent in the target quadrant was recorded by means of computerized video tracking.

*Three-chamber social behavior test*

In the rectangular three-chamber apparatus, the central chamber and two side-chambers, 19cm x 45cm each, were separated by clear Plexiglas walls. Openings between the chambers allowed the test mouse free access to all the chambers, and a cup-like container placed inside each side-chamber was available for holding a stranger mouse^6^. Prior to experiment, test mouse was placed in the center of the middle chamber and given 5 minutes of habituation time. This was followed by a 10-minute social affiliation (SA) test, when a Stranger-1 mouse of the same age, with no previous contact with test mouse, was placed into the cup-like container in one of the side chambers, and the number and duration of close contacts initiated by the test mouse with Stranger-1 were recorded in order to determine the extent of social affiliation displayed by the test mouse. Upon completion of the SA test, a 10-minute social-novelty preference (SN) test was conducted, when another same-age Stranger-2 mouse, also with no previous contact with test mouse, was placed into the cup-like container in the other side chamber. Since the original Stranger-1 mouse was already known to the test mouse, it in effect became a Familiar-1 mouse to the test mouse. During this SN test, the number and duration of close contacts initiated by the test mouse toward the Stranger-2 and Familiar-1 mice were recorded separately and compared in order to determine the extent of social-novelty preference displayed by the test mouse.

*Tail suspension test*

The tail suspension apparatus, with dimensions of 40cm x 40cm x 60cm, consisted of a plastic box with a horizontal bar set 15 cm above the floor. The test mouse was taped by the tail to the bar and remained suspended for a 6-min test period. Its immobility time between minute-2 and minute-6 was recorded^7^.

*Sucrose preference test*

Each test mouse was individually housed and tested in its home cage. Prior to the test, it was habituated with a 2-bottle liquid-supply setting, with water in both bottles, for 48 hrs. This was followed by the actual test: one bottle contained 2% sucrose solution and the other contained water during the first 24 hours, and the two bottles were switched during the next 24 hours^8^. The amounts of sucrose solution and water consumed during the entire 48-hour test period were recorded in order to determine the % sucrose preference, which was expressed as the percentage of sucrose solution consumed relative to total liquid consumption.

*Elevated plus-maze test*

The test apparatus comprised two opposite open arms and two opposite closed arms, each of 25cm x 5cm. The four arms extended from a central 5cm x 5cm platform in the shape of a plus sign. The closed arms were enclosed by 20 cm high opaque walls and the maze was raised 40 cm above ground^2^. Test mouse was placed at the center of the maze facing an open arm, and allowed to roam and explore the maze for 5 minutes. The number of entries and time spent in the open arms during this period were recorded, and expressed as percentage of total number of arm entries and percentage of total time spent in all the arms, respectively.

*Holeboard test*

The holeboard apparatus had dimensions of 60 x 60 x 20 cm. There were four holes of 3 cm diameter each evenly distributed on the floor. At the start of the experiment, the mouse was was placed at the center of the apparatus, and the number of rearing and head-dips into the holes were counted over a 5-minute period^2^.

*Audiogenic epilepsy and pentylenetetrazol-induced seizure*

The audiogenic seizure test protocol was similar to that previously described^9^. The apparatus employed consisted of a plastic chamber (30cm x 20cm x 30cm) with an alarm (Kerui, Model KR-X2; Kerui, Shenzhen, PRC) attached to the ceiling. Each test mouse was habituated individually in the chamber for 60s. White noise stimulus (110dB) was generated by an alarm and maintained until the onset of seizure or up to a period of 60s. Possible motor responses to the noise stimulus, including no response, wild running and tonic seizure, were recorded. Susceptibility to drug-induced seizure was tested by PTZ injection (100 mg/kg, i.p.; Sigma-Aldrich, USA). Each PTZ-treated mouse was individually placed in the plastic chamber for a 10-minute observation period, and the latency to seizure onset was recorded^10^.

**Immunohistochemical analysis**

The protocol employed was similar to that described previously^11^. The mouse was anesthetized and perfused intracardially with saline, followed by 4 % (w/v) paraformaldehyde in 0.1 mol/L PBS, pH7.4. Brain was removed and fixed overnight in 4 % (w/v) paraformaldehyde, then transferred to 25 % sucrose in PBS and kept in that solution until it sank to the bottom. Thereafter, the tissue block was rapidly frozen and coronal sections (20 μm in thickness) were cut with a Leica cryostat and floated in PBS. The sections were washed 3–5 min in PBS, and then pre-incubated in a blocking solution (10 % normal bovine serum, 0.2 % Triton X-100, 0.4 % sodium azide in 0.01 mol/l PBS pH 7.2) for 30 min followed by incubation with the primary antibodies against NeuN (1:500, monoclonal, clone A60; EMD Millipore, MA), GFAP (1:500, rabbit polyclonal; Boster Biological Technology, CA), DCX (1:200, goat polyclonal; Santa Cruz Biotechnology, TX), Iba1 (1:500, rabbit polyclonal; Wako, Japan), and parvalbumin (PV, 1:3000, rabbit polycolonal, Proteintech, IL) at room temperature overnight. Subsequently the sections were incubated with FITC-conjugated donkey anti-mouse IgG (Jackson ImmunoResearch Inc., PA) diluted 1:200 for NeuN; Cy3-conjugated donkey anti-rabbit IgG (Jackson ImmunoResearch Inc., PA) diluted 1:400 for GFAP and Iba1; FITC-conjugated donkey anti-rabbit IgG (Jackson ImmunoResearch Inc., PA) diluted 1:400 for PV; and Cy3-conjugated donkey anti-goat IgG (Jackson ImmunoResearch Inc., PA) diluted 1:400 for DCX. All serial incubations were separated by five 10-min washes with PBS. Finally, the sections were mounted and covered with 50% glycerin PBS.

Images were taken with a Nikon digital camera DXM1200 (Nikon, Japan) attached to a Nikon Eclipse E600 microscope, and imported into a graphics package (Adobe Photoshop 5.0, USA). The number of cells immunostained with NeuN, GFAP, PV, DCX and Iba1 antibodies in the detected regions were counted under 20x objective lens and analyzed using the NIS-elements D3.1 system (Nikon, Japan). Five sections were used for each mouse and the mean number of neurons was calculated. The immunofluorescence optical density (O.D.) for images from the regions stained with antibodies against NeuN, GFAP and PV were also determined using the NIS-elements D3.1 system. Five images (100×) were randomly selected for each mouse to yield the mean O.D., and statistical analysis was performed for five mice per group.

**SUPPLEMENTARY REFERENCES**

1. Li, Q. et al. Prenatal immune challenge is an environmental risk factor for brain and behavior change relevant to schizophrenia: evidence from MRI in a mouse model. *PLoS One* **4**, e6354 (2009).

2. Hui, K. M. et al. Anxiolytic effect of wogonin, a benzodiazepine receptor ligand isolated from Scutellaria baicalensis Georgi. *Biochem. Pharmacol.* **64**, 1415-1424 (2002).

3. Peça, J. et al. Shank3 mutant mice display autistic-like behaviours and striatal dysfunction. *Nature* **472**, 437-442 (2011).

4. Dellu, F., Contarino, A., Simon, H., Koob, G. F. & Gold, L. H. Genetic differences in response to novelty and spatial memory using a two-trial recognition task in mice. *Neurobiol. Learn. Mem.* **73**, 31-48 (2000).

5. Xu, J., Zhu, Y., Contractor, A. & Heinemann, S. F. mGluR5 has a critical role in inhibitory learning. *J. Neurosci.* **29**, 3676-3684 (2009).

6. Park, S. J. et al. Toll-like receptor-2 deficiency induces schizophrenia-like behaviors in mice. *Scientific Reports* **5**, 8502 (2015).

7. Lu, X., Ross, B., Sanchez-Alavez, M., Zorrilla, E. P. & Bartfai, T. Phenotypic analysis of GalR2 knockout mice in anxiety- and depression-related behavioral tests. *Neuropeptides* **42**, 387-397 (2008).

8. Koo, J. W. & Duman, R. S. IL-1beta is an essential mediator of the antineurogenic and anhedonic effects of stress. *Proc. Natl. Acad. Sci. USA* **105**, 751-756 (2008).

9. Venit, E. L., Shepard, B. D. & Seyfried, T. N. Oxygenation prevents sudden death in seizure-prone mice. *Epilepsia* **45**, 993-996 (2004).

10. Ren, L. et al. GABA(A) receptor subtype selectivity underlying anxiolytic effect of 6-hydroxyflavone. *Biochem. Pharmacol.* **79**, 1337-1344 (2010).

11. Ge, Y. et al. Intrathecal infusion of hydrogen-rich normal saline attenuates neuropathic pain via inhibition of activation of spinal astrocytes and microglia in rats. *PLoS One* **9**, e97436 (2014).

**SUPPLEMENTARY FIGURES**

**
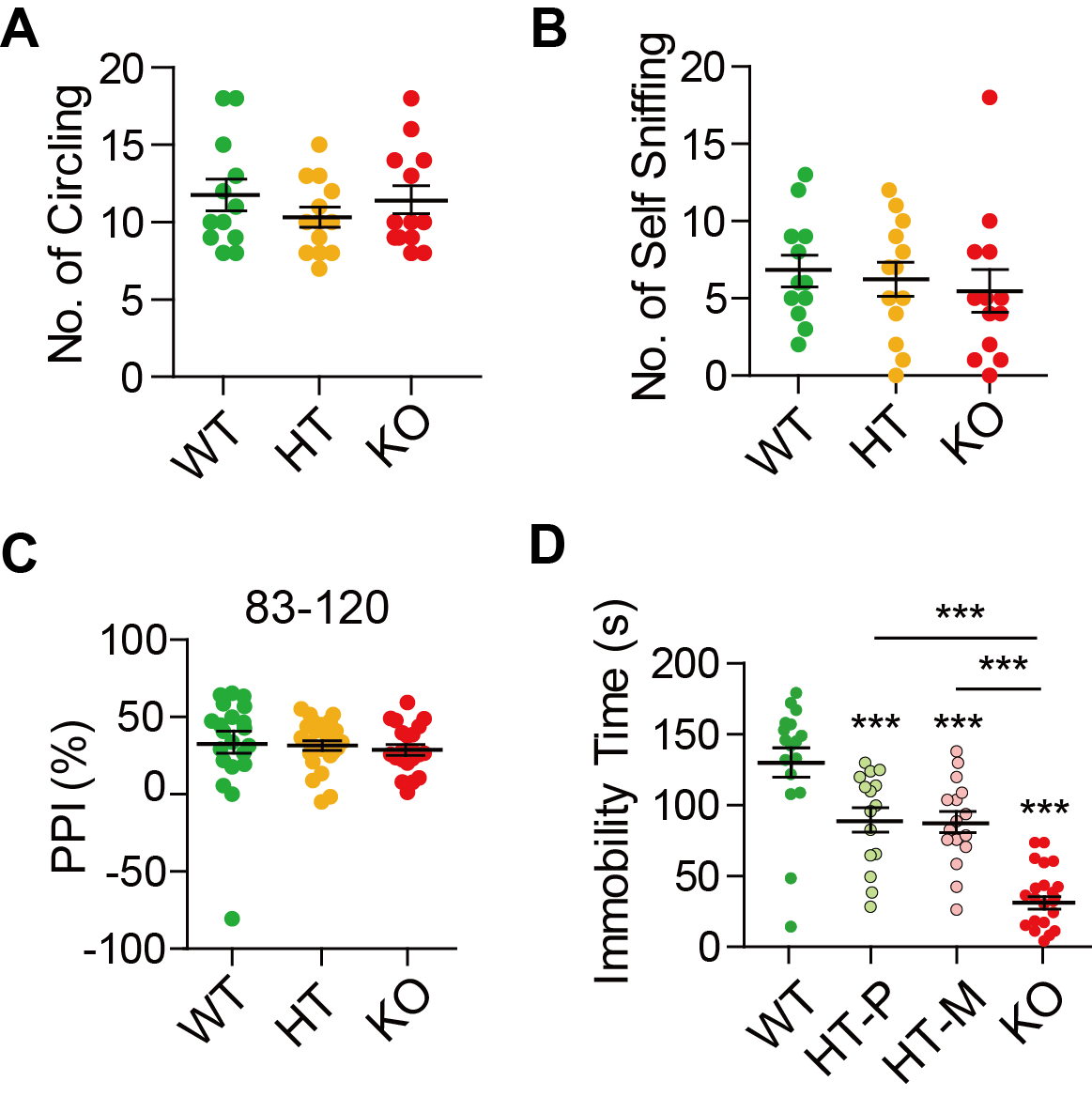
**

**Supplementary Figure S1. Neuropsychiatric behavior.** (a,b) Behavioral stereotypy. The numbers of (a) circling and (b) self-sniffing monitored during a 5-min period (WT male *n*=12, HT male *n*=13, KO male *n*=13). (c) Percentile PPI in 83-120 dB prepulse-pulse trials (WT male *n*=21, HT male *n*=25, KO male *n*=21). (d) Behavioral genetic imprinting: no significant difference in immobility time was observed between HT-P and HT-M in the tail-suspension test (WT male *n*=17, HT-P male *n*=15, HT-M male *n*=16, KO male *n*=22). Statistical analysis was performed using one-way ANOVA with Newman Keuls post-hoc test (*** *p* < 0.001). WT is represented by green dots, HT by orange dots, KO by red dots, HT-P by light green dots, and HT-M by pink dots.


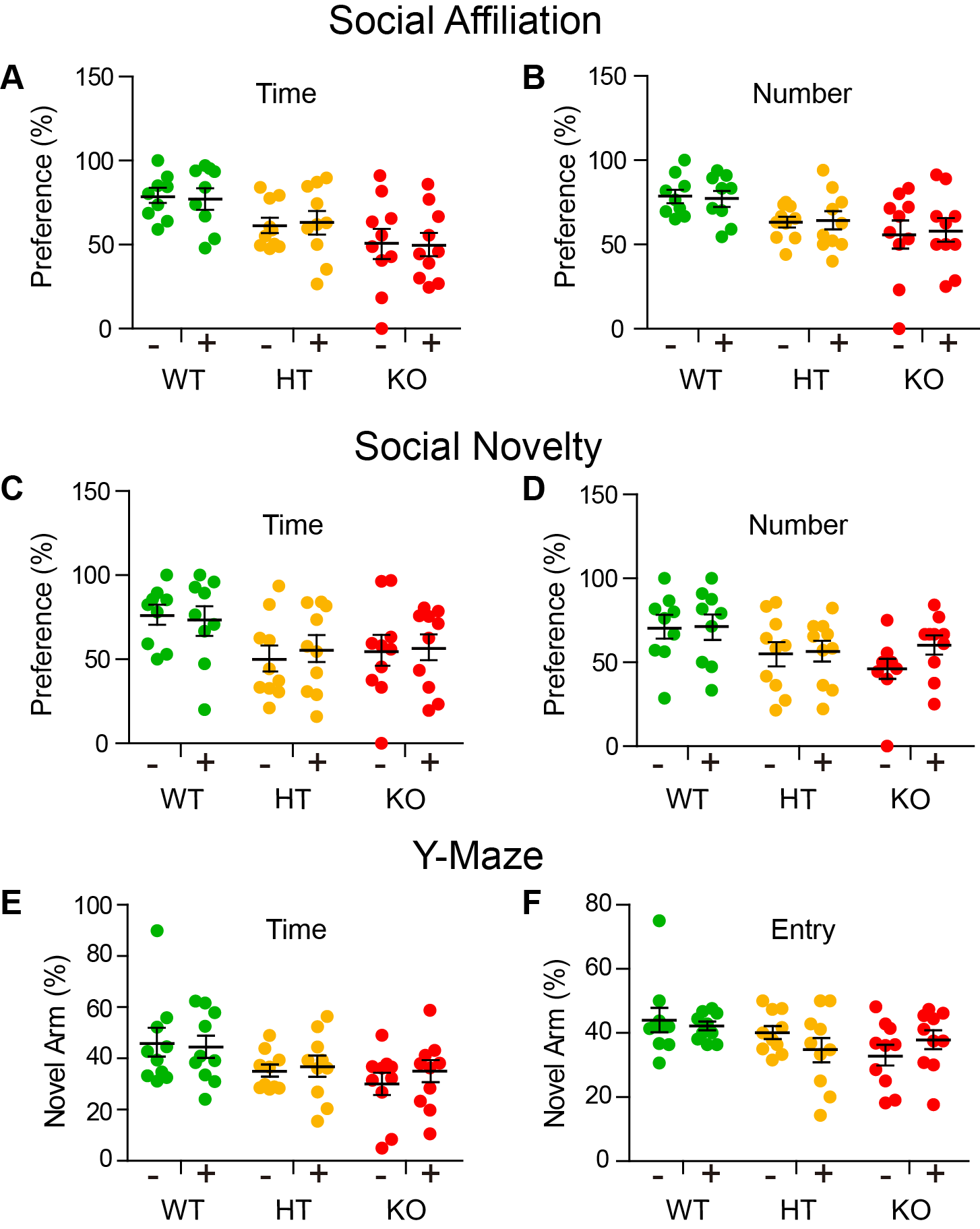


**Supplementary Figure S2. Risperidone effect on social interaction and cognition.** (a,b) Social affiliation test; and (c,d) Preference for social novelty test (saline group: WT female *n*=9, HT female *n*=10, KO female *n*=10; risperidone group: WT female *n*=9, HT female *n*=10, KO female *n*=10). Preference for container holding Stranger-1 mouse relative to empty container was monitored based on time of visitations in (a), or based on number of visitations in (b). Preference for container holding Stranger-2 mouse relative to container holding Familiar-1 mouse was monitored based on time of visitations in (c), or based on number of visitations in (d). (Saline group: WT male *n*=10, HT male *n*=10, KO male *n*=10; risperidone group: WT male *n*=10, HT male *n*=10, KO male *n*=10). (e,f) Y-maze test showing time spent, or entries into, novel arm. Statistical analysis was performed using two-way ANOVA with Dunnett's post-hoc test. WT is represented by green dots, HT by orange dots, and KO by red dots. Animals administered with 0.3 mg/kg risperidone i.p. are denoted by ‘+’, and animals administered with saline i.p. are denoted by ‘-’. There was no detectible significant difference with *p* < 0.05 in the plots.


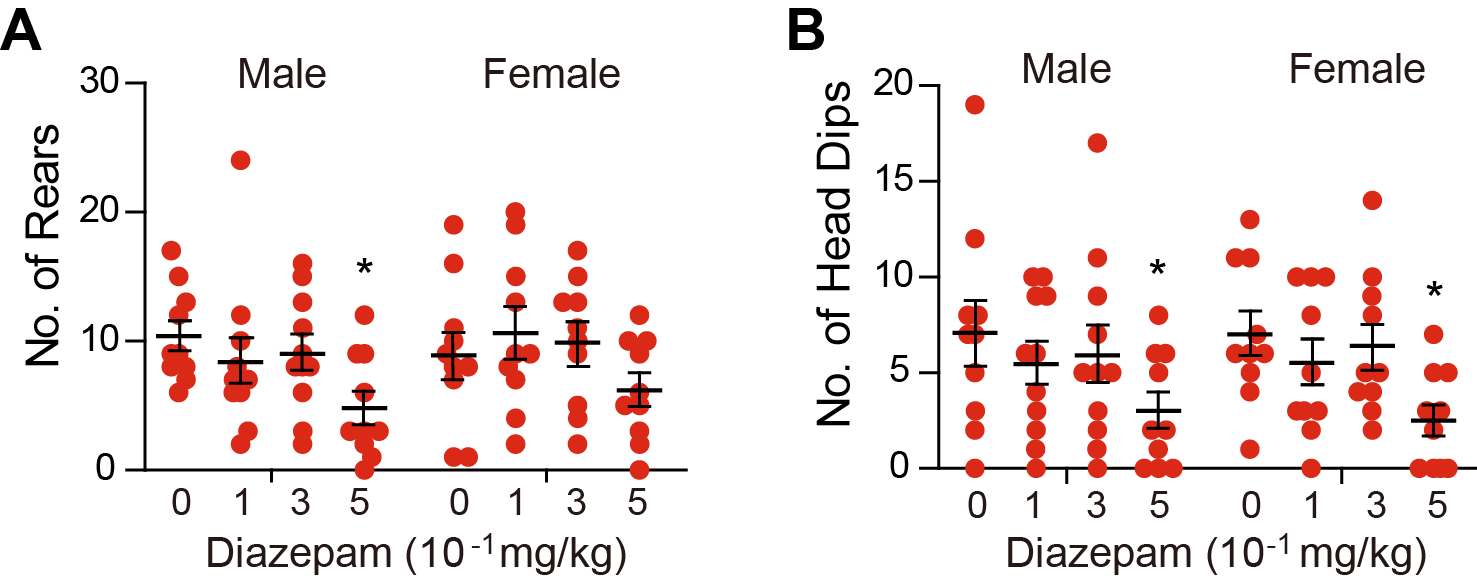


**Supplementary Figure S3. Sedative effect of diazepam on KO mice.** Sedation was monitored for male and female KO mice based on the numbers of rears and head-dips in the holeboard test (saline group: WT male *n*=10, KO male *n*=10; 0.1mg/kg risperidone group: WT male *n*=11, KO male *n*=10; 0.3mg/kg risperidone group: WT male *n*=10, KO male *n*=10; 0.5mg/kg risperidone group: WT male *n*=10, KO male *n*=10). Statistical analysis was performed using one-way ANOVA with Newman Keuls post-hoc test (* *p* < 0.05).


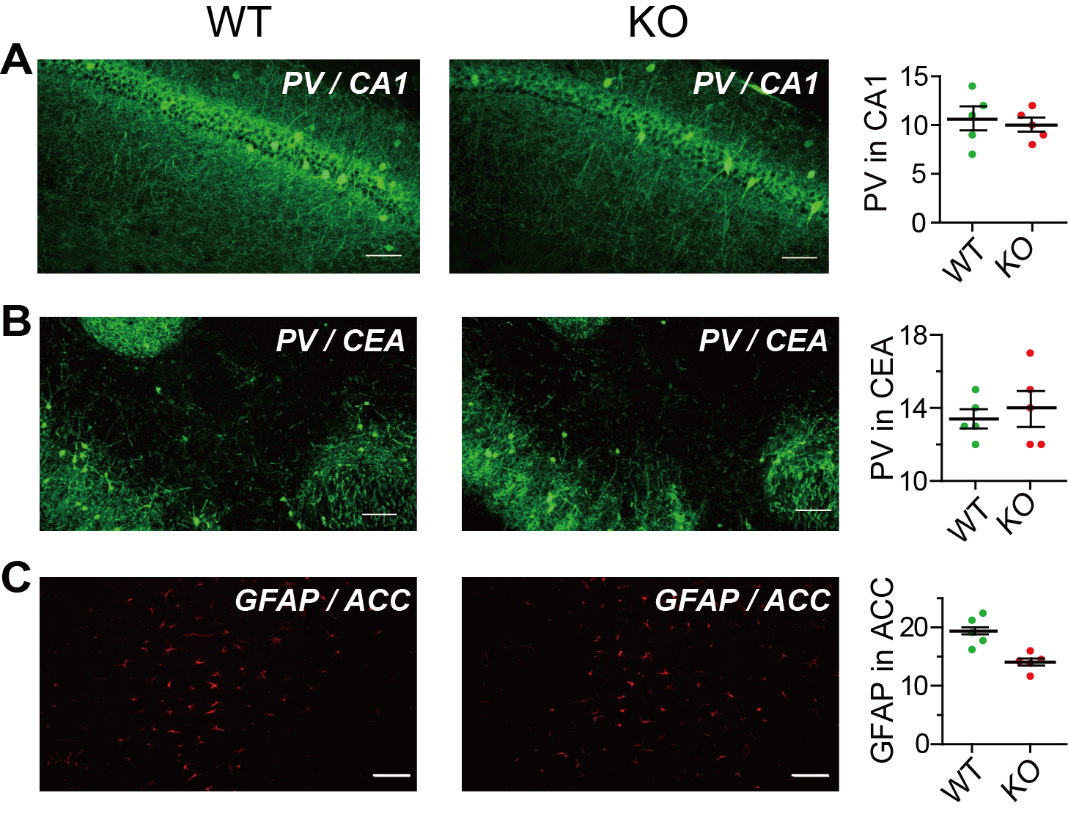


**Supplementary Figure S4. Immunohistochemical staining of mouse brain.** Coronal sections were stained with fluorescence-labelled antibodies for (a) parvalbumin (PV) in CA1 of hippocampus; (b) PV in central amygdaloid nucleus (CEA); and (c) GFAP in anterior cingulate cortex (ACC). (WT and KO male *n*=5 per group). The numbers of PV-positive neurons in (a) and (b), and the immunofluorescence optical densities of GFAP in (c), were in each instance not significantly different between WT and KO mice. Statistical analysis was performed using unpaired *t*-test. WT is represented by green dots, and KO by red dots. There was no detectible significant difference with *p* < 0.05 in the plots.


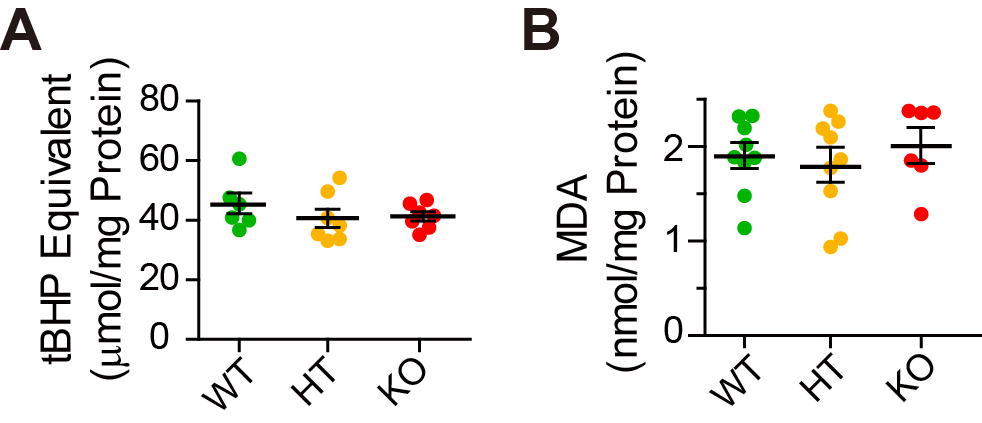


**Supplementary Figure S5. Markers of oxidative stress in mouse blood and liver.** Oxidative stress in mice was determined as levels of (a) reactive oxygen metabolites in terms of tert-butylhydroperoxide (tBHP) equivalents in blood (WT male *n*=6, HT male *n*=7, KO male *n*=7); and (b) malondialdehyde (MDA) in liver (WT male *n*=9, HT male *n*=9, KO male *n*=6). Statistical analysis was performed using one-way ANOVA with Newman Keuls post-hoc test (* *p* < 0.05). WT is represented by green dots, HT by orange dots, and KO by red dots. There was no detectible significant difference with *p* < 0.05 in the plots.

**SUPPLEMENTARY TABLES**

**Supplementary Table S1. Primer sequences**

|  | Gene | Forward Primer (5’ to 3’) | Reverse Primer (5’ to 3’) |
| --- | --- | --- | --- |
| (i) | *Gabrb2* (Taconic) | TGTTTCCTCTTACAGATGGCTACAC | CCTGTGGAGAAAACAACTTTCTTG |
|  | *Neo* (Taconic) | GATGGATTGCACGCAGGTTCT | AGGTAGCCGGATCAAGCGTAT |
| (ii) | *Gabrb2* (exon 1) | GTCTGGATCTGGTGTCCGTG | AGGCACAATGAAGGAACCCC |
| (iii) | *Gabra1* | AAAAGCGTGGTTCCAGAAAA | GCTGGTTGCTGTAGGAGCAT |
|  | *Gabra2* | GCTACGCTTACACAACCTCAGA | GACTGGCCCAGCAAATCATACT |
|  | *Gabra3* | GCCGTCTGTTATGCCTTTGTATTT | TTCTTCATCTCCAGGGCCTCT |
|  | *Gabra4* | AGAACTCAAAGGACGAGAAATTGT | TTCACTTCTGTAACAGGACCCC |
|  | *Gabra5* | GATTGTGTTCCCCATCTTGTTTGGC | TTACTTTGGAGAGGTGGCCCCTTTT |
|  | *Gabra6* | GGTGACCGGGCATCCCAGTGA | TGTTACAGCACCCCCAAATCCTGGC |
|  | *Gabrb1* | GGTTTGTTGTGCACACAGCTCC | ATGCTGGCGACATCGATCCGC |
|  | *Gabrb2* (exon 7) | GCTGGTGAGGAAATCTCGGTCCC | CATGCGCACGGCGTACCAAA |
|  | *Gabrb3* | GAGCGTAACGACCCCGGGAA | GGGACCCCCGAAGTCGGGTCT |
|  | *Gabrg1* | ATCCACTCTCATTCCCATGAACAGC | ACAGAAAAAGCTAGTACAGTCTTTGC |
|  | *Gabrg2* | ACTTCTGGTGACTATGTGGTGAT | GGCAGGAACAGCATCCTTATTG |
|  | *Gabrg3* | AATACATCCAGATTCCACAAGATG | CACAGGTGTCCTCAAATTCCT |
|  | *Gabrd* | CCACTTCAATGCCGACTACA | TGAGAGGGAGAAAAGGACGA |
|  | *Actb* | CTTCTTTGCAGCTCCTTCGT | GATGGAGGGGAATACAGCCC |
|  | *Pgk1* | CTGACTTTGGACAAGCTGGACG | GCAGCCTTGATCCTTTGGTTG |

Primer sequences used for (i) determining *Gabrb2* genotype in mice; (ii) confirming absence of *Gabrb2* expression in KO mice; and (iii) quantitating GABA_A_ receptor subunit mRNA expressions in mice using the QuantiTect® reverse transcription kit (Qiagen).

**Supplementary Table S2. Average litter size per mother delivered by naive 8-week old breeders** ^a^

|  |  |  | Female |  |
| --- | --- | --- | --- | --- |
|  | Genotype | KO | HT | WT |
|  | KO | 3.7 ^b^ | 5.4 | 5.6 |
| Male | HT | 6.3 | 6.5 | 6.6 |
|  | WT | 6.4 | 6.5 | 6.3 |

^a^ *n* = 4-7 cages; 1 male and 2 female breeders per cage

^b^ Statistically significant decrease compared to WT/WT pairs (*p* < 0.05; unpaired *t*-test).

| Age | DZ (mg/kg)^b^ | Gender | Prevalence | % | Death rate | % |
| --- | --- | --- | --- | --- | --- | --- |
| week 3 | 0 | Male | 18/19 | 95 | 3/18 | 17 |
|  | 0 | Female | 19/20 | 95 | 1/19 | 5 |
| week 10 | 0 | Male | 16/16 | 100 | 1/16 | 6 |
|  | 0.1 | Male | 9/11 | 82 | 1/9 | 11 |
|  | 0.3 | Male | 6/10 | 60 | 0/6 | 0 |
|  | 0.5 | Male | 3/10 | 30 | 0/3 | 0 |
|  | 0 | Female | 16/16 | 100 | 1/16 | 6 |
|  | 0.1 | Female | 8/11 | 73 | 0/8 | 0 |
|  | 0.3 | Female | 6/10 | 60 | 0/6 | 0 |
|  | 0.5 | Female | 4/10 | 40 | 0/4 | 0 |

**Supplementary Table S3. Prevalence of seizure and death rate of audiogenic epilepsy in *Gabrb2* KO mice ^a^**

^a^ *n* = 10-20 per group

^b^ Diazepam (DZ) administered i.p.

**Supplementary Table S4. Relative amounts of different GABA_A_ receptor subunit mRNAs in mouse brain**

|  | Cerebrum | | | Cerebellum | | |  |
| --- | --- | --- | --- | --- | --- | --- | --- |
|  | WT | HT | KO | WT | HT | KO | |
| *Gabra1* | 1.483±0.053 | 1.459±0.059 | **1.146±0.068** (**) | 1.390±0.086 | **1.090±0.091** (**) | **0.870±0.041** (***) | |
| *Gabra2* | 0.334±0.027 | **0.271±0.016 (*)** | **0.234±0.014** (**) | 0.174±0.023 | **0.123±0.010** (*) | **0.105±0.009** (**) | |
| *Gabra3* | 0.110±0.006 | 0.110±0.006 | 0.114±0.005 | 0.105±0.010 | 0.117±0.007 | 0.111±0.013 | |
| *Gabra4* | 0.209±0.008 | 0.233±0.010 | 0.196±0.006 | 0.131±0.012 | 0.109±0.009 | 0.129±0.013 | |
| *Gabra5* | 0.728±0.040 | **0.513±0.059** (*) | **0.558±0.056** (*) | 0.109±0.014 | 0.085±0.014 | **0.054±0.006** (**) | |
| *Gabra6* | N.D. | N.D. | N.D. | 2.089±0.109 | **1.647±0.121** (*) | **1.594±0.123** (*) | |
| *Gabrb1* | 0.327±0.012 | 0.317±0.031 | ***0.486±0.051*** (**) | 0.156±0.010 | 0.175±0.026 | 0.187±0.019 | |
| *Gabrb2* | 0.057±0.004 | **0.043±0.004** (*) | N.D. | 0.051±0.004 | **0.032±0.002** (**) | N.D. | |
| *Gabrb3* | 0.282±0.026 | 0.291±0.020 | 0.335±0.039 | 0.101±0.005 | 0.105±0.013 | 0.121±0.012 | |
| *Gabrg1* | 0.128±0.006 | 0.140±0.013 | ***0.229±0.038*** (*) | 0.078±0.008 | 0.085±0.013 | 0.111±0.013 | |
| *Gabrg2* | 0.714±0.021 | 0.913±0.134 | ***1.158±0.127*** (*) | 0.904±0.048 | 0.991±0.077 | 1.032±0.091 | |
| *Gabrg3* | 0.047±0.003 | 0.044±0.006 | 0.047±0.006 | 0.024±0.001 | 0.024±0.003 | 0.022±0.001 | |
| *Gabrd* | 0.034±0.010 | 0.042±0.019 | 0.049±0.014 | 0.096±0.028 | 0.110±0.027 | ***0.133±0.031*** (*) | |

Levels of mRNAs were determined relative to mRNAs of *Actb* and *Pgk1*. Levels in HT and KO that were significantly altered compared to WT are shown in bold font, in italics for increases or non-italics for decreases. * *p* < 0.05; ** *p* < 0.01; *** *p* < 0.001; N.D. indicates non-detectable. For *Gabrb2*, mRNA was determined using exon 7-specific primer sequences (Supplementary Table S1).
